# Study of the Arrhythmogenic Profile in Dogs with Acute and Chronic Monocytic Ehrlichiosis

**DOI:** 10.3390/life15030490

**Published:** 2025-03-18

**Authors:** Carolina Dragone Latini, Angélica Alfonso, Maurício Gianfrancesco Filippi, Mayra de Castro Ferreira Lima, Antônio Carlos Paes, Jaqueline Valença Corrêa, Beatriz Almeida Santos, Miriam Harumi Tsunemi, Maria Lucia Gomes Lourenço

**Affiliations:** 1 School of Veterinary Medicine and Animal Science, São Paulo State University (UNESP), Botucatu 18618-681, Brazil; carolina.latini@unesp.br (C.D.L.); alfonso_angelica@ymail.com (A.A.); mauriciofilippi@terra.com.br (M.G.F.); mayracfl@hotmail.com (M.d.C.F.L.); ac.paes@unesp.br (A.C.P.); jaqueline.v.correa@unesp.br (J.V.C.); beatriz.a.santos@unesp.br (B.A.S.); 2 Institute of Biosciences, São Paulo State University (UNESP), Botucatu 18618-691, Brazil; m.tsunemi@unesp.br

**Keywords:** arrhythmias, canine hemoparasitosis, electrocardiogram, Holter, arrhythmogenesis

## Abstract

Canine monocytic ehrlichiosis (CME) is a globally prevalent disease transmitted by the tick *Rhipicephalus sanguineus* and caused by the Gram-negative bacterium *Ehrlichia* spp. Following an incubation period, the infection is categorized based on the progression of the disease into acute, subclinical, and chronic stages. Besides hematological alterations, the cardiovascular system is significantly impacted by the hemodynamic effects of the disease, as persistent anemia can lead to myocardial hypoxia and the activation of inflammatory processes, potentially causing myocarditis. It is known that in dogs infected with *Ehrlichia canis*, there is a higher occurrence of arrhythmias and a predominance of sympathetic activity. This study assessed arrhythmogenic parameters, including P wave dispersion (Pd), QT dispersion (QTd), and QT instability, along with heart rate variability (HRV) analysis from 24 h Holter monitoring in naturally infected dogs during the acute phase (*n* = 10) and chronic phase (*n* = 10) compared to a control group (*n* = 10). The Pd and QTd values were higher in the infect group, confirming the arrhythmogenic character. Instability parameters (TI, LTI, and STI) were higher in sick animals, but no worsening was observed in the chronic phase. All HRV metrics in the time domain were higher in the control group, indicating a balanced sympathovagal activity throughout the day in healthy dogs. Additionally, parameters linked to parasympathetic activity (rMSSD and pNN50) were reduced in the sick groups, confirming the dominance of sympathetic activity. These findings indicate a decrease in HRV in sick individuals and reinforce this useful marker for assessing the influence of the autonomic nervous system on the cardiovascular system. In conclusion, CME exhibits arrhythmogenic activity characterized by the deterioration of predictive parameters for ventricular arrhythmias and increased activity of the sympathetic autonomic nervous system in the heart. This is likely secondary to myocarditis, myocardial hypoxia, and structural damage to cardiomyocytes.

## 1. Introduction

Canine monocytic ehrlichiosis (CME) is a worldwide disease caused by the bacterium *Ehrlichia canis* and transmitted by the Rhipicephalus sanguineus tick [1]. The genus Ehrlichia consists of obligate intracellular Gram-negative bacteria that are pleomorphic and primarily infect mononuclear cells, resulting in the formation of intracytoplasmic structures called mulberries. Although six other species of Ehrlichia are known, *E. canis* was the first to be identified in dogs and is the leading cause of CME [2].

After an incubation period of one to three weeks, the disease progresses through three successive phases: acute, subclinical, and chronic. The acute phase usually lasts two to four weeks, but symptoms vary from person to person, and some may remain asymptomatic. Particularly in asymptomatic patients, the disease may improve without treatment, and they may remain subclinical carriers for months or even years, which has significant epidemiological implications. During the subclinical stage, dogs do not display overt symptoms but may show hematologic changes such as thrombocytopenia and hyperglobulinemia. In some cases, patients may progress to a chronic phase with worsening disease and an increased likelihood of complications [3]. Cardiac abnormalities have been observed in dogs infected with *E. canis*, classifying this disease as a possible cause of cardiomyopathy associated with myocardial damage [4]. The cardiac changes seen in hemoparasitosis diseases are due to persistent anemia, which, depending on the degree, developmental timing, and hemodynamic adaptations, may lead to hypoxia and structural changes in myocytes. Activation of the inflammatory process that occurs during the disease can lead to myocarditis and subsequently to supraventricular and ventricular arrhythmias. Therefore, the electrocardiogram is an important diagnostic tool for identifying arrhythmias and evaluating changes suggestive of myocardial hypoxia [5].

Based on the available literature, CME has been suggested as a possible cause of canine myocarditis. The likelihood of arrhythmic events increases both in the acute and chronic phases of the disease, with a greater impact in the chronic phase. In addition, a predominant activation of the sympathetic nervous system has been observed [6,7]. The aim of this study was to investigate the arrhythmogenic features of dogs infected with *Ehrlichia canis* during both the acute and chronic phases. Predictive parameters for the occurrence of arrhythmias, including P wave dispersion, QT variability, and dispersion, were examined, while heart rate variability was analyzed in comparison with healthy animals.

## 2. Materials and Methods

### 2.1. Animals

Thirty dogs were divided into three groups of ten dogs each: (G1) dogs diagnosed with acute mononuclear ehrlichiosis; (G2) dogs diagnosed with chronic mononuclear ehrlichiosis; and (G3) a control group.

The dogs were included in the services provided by the Infectious Diseases Diagnostic Unit, Department of Hygiene and Public Health, Faculty of Veterinary Medicine and Animal Husbandry, State University of Botucatu Paulista “Júlio de Mesquita Filho” (UNESP), São Paulo, Brazil, through a retrospective analysis conducted between July 2014 and July 2016. The dogs in the control group were selected between August 2020 and August 2022 and accepted to participate in this study after an initial evaluation.

The inclusion criteria for the G1 and G2 groups were as follows: (1) clinical changes consistent with the medical history, (2) systemic circulation pressure between 110 and 140 mmHg, and (3) hematological changes consistent with the respective disease stages (G1: <160,000 platelets/μL; GS: glomerular volume <25%, <3000 leukocytes/μL, <20,000 platelets/μL), and a positive *Ehrlichia canis* PCR.

Criteria for inclusion of animals in the G3 (control group) were (1) systemic arterial pressure between 110 and 140 mmHg during all measurements; (2) no changes in the physical examination; and (3) maintenance of a previously confirmed health history by the caregiver, including no history of canine mononuclear Ehrlichia.

The exclusion criteria for the medical group included the presence of a heart murmur on auscultation or previous heart disease, cardiovascular resuscitation within the past 90 days, and previous treatment with antibiotics for *Ehrlichia canis* (e.g., tetracycline). For the G2 group, the dogs had to be less than five years old. This study was approved by the Ethical Committee for the Use of Animals–CEUA–FMVZ–UNESP, Botucatu, São Paulo, Brazil, with protocol number 0194/2022, to ensure compliance with ethical standards for animal experimentation. Participation in this study required consent from the owner, who signed a consent form.

### 2.2. Systemic Blood Pressure Measurement

First, systemic blood pressure was measured using a noninvasive method to minimize external factors that could increase blood pressure values, such as stress. The device used for this purpose was a Parks Vascular Doppler device model 811-B (Parks Medical, Aloha, OR, USA).

The cuff was selected based on the circumference of the dog’s leg, specifically, 40% of the leg diameter, and was placed on the distal radial portion of the left foreleg. Three measurements were recorded, and the final value was calculated as the arithmetic mean of these three values. According to the consensus of the American College of Veterinary Internal Medicine, a normotensive patient was one whose systolic blood pressure for the three measurements and the final mean was between 120 and 139 mmHg [8].

### 2.3. Collection and Analysis of Biological Material

Ten milliliters of blood were drawn from each animal in the diseased group by jugular venipuncture. The blood was divided into two tubes: one tube contained the anticoagulant EDTA-K2 for complete blood count and molecular testing, while the rest was placed in a tube containing a coagulation activator for serum biochemistry analysis (including urea, creatinine, ALT, AST, FA, total protein, and albumin) for diagnostic purposes.

The molecular test used was polymerase chain reaction (PCR), and the samples were sent to a laboratory (VetDNA) in Botucatu, São Paulo, Brazil. The methods used were described in detail in a previous study [9].

### 2.4. Conventional Electrocardiogram

ECG recordings were performed for five minutes using a 6-channel electrocardiograph (ECGPCVet, TEB) with bipolar (I, II, III) and unipolar (aVR, aVL, and aVF) leads at a speed of 25 mm/s and a sensitivity of 1 cm = 1 mV [10,11]. The patients were placed in the right lateral decubitus position, and no sedation was given. Electrodes were constructed according to the procedure described by Santilli et al. [11] and placed and prepared with a 70% alcohol solution. Wave duration and amplitude analysis was performed using a bipolar DII wire.

To determine wave dispersion, the duration of the P wave from the beginning to the end in all leads was measured and recorded in milliseconds. The minimum P wave value (Pmin) and the maximum P wave value (Pmax) were determined for each lead. P wave dispersion (Pd) was calculated as the difference between Pmax and Pmin (Pd = Pmax − Pmin). The arithmetic mean was derived from three different cardiac cycles.

The QT dispersion was measured in each lead, and the duration of the QT interval (in seconds) occurring from the beginning of the QRS complex to the end of the T wave was recorded. The minimum QT interval value (QTmin) and the maximum QT interval value (QTmax) were determined for each lead. The QT dispersion (QTd) was calculated as the difference between QTmax and QTmin (QTd = QTmax − QTmin). The center of mass and rotation center of mass were calculated based on the previous measurements, and the total instability (TI), long-term instability (LTI), and short-term instability (STI) were determined, in turn, according to the model proposed in the literature [12].

The arithmetic mean was calculated from three different cardiac cycles. Based on the QT measurement results, the formula suggested by Fridericia [13] was applied for correction:

$$\mathrm{QTc}=\frac{\mathrm{QT}}{{\mathrm{HR}}_{3}^{1}}$$

where QTc is the corrected QT; QT is the measured QT interval; and HR is the heart rate. QTm (mean QT) and QTv (variance QT) were then calculated for QT and QTc.

### 2.5. Ambulatory Electrocardiogram (Holter)

Holter monitoring data were collected using the digital three-channel device Cardiolight (Cardios, São Paulo, Brazil). Four electrodes were placed horizontally on both sides of the chest between the third and fifth intercostal spaces, and the acquired data were automatically analyzed by the Cardiosmart 550 device (Cardios, São Paulo, Brazil). Manual corrections were performed when necessary. The main parameters evaluated included basal rhythm, minimum, mean, and maximum heart rates, pauses longer than two seconds, the presence or absence of abnormalities, and supraventricular and ventricular ectopic events. The acceptable artifact threshold was set to less than 5% (*p* < 0.05).

### 2.6. Statistical Analysis

The Shapiro–Wilk test and Levene’s test for homogeneity of variance were used to assess the assumption of normality in the distribution of the quantitative variables in the healthy dogs and dogs with CME in the acute and chronic phases.

For intergroup comparisons, ANOVA was applied to variables that met the assumption of normality. Conversely, the Kruskal–Wallis test was used for variables that did not meet this assumption. Dunn’s post hoc test was also performed. Qualitative variables were assessed using the chi-square test. Within each group, Pd, QTd, TI, STI, and LTI were compared with heart rate (HR) and age using Pearson and Spearman correlation analyses. The same method was used to analyze the relationship between Pd, QTd, TI, STI, and LTI and parameters determined by Holter, including heart rate variability (HRV) in the time domain, standard deviation of normal RR or NN intervals over a period of time (SDNN), standard deviation of the mean of normal R-R intervals per 5 min (SDANN), root mean square of consecutive differences in consecutive R-R intervals (rMSSD), and percentage of adjacent R-R intervals with a duration difference of more than 50 ms (pNN50), as well as HRV in the frequency domain [high-frequency component (HF), low-frequency component (LF), low-frequency/high-frequency ratio (LF/HF), and very low frequency (VLF)]. All analyses were performed at a significance level of 5% (*p* < 0.05).

## 3. Results

The study sample included 30 animals of different breeds and sexes (40% females, *n* = 12/30, 60% males, *n* = 18/30). The group included mixed breed dogs (*n* = 12/30; 40%), Yorkshire terriers (*n* = 3/30; 10%), Labrador retrievers (*n* = 2/30; 6.6%), poodles (*n* = 2/30; 6.6%), pugs (*n* = 2/30; 6.6%), a Lhasa apso (*n* = 1/30; 3.3%), a pit bull (*n* = 1/30; 3.3%), a French bulldog (*n* = 1/30; 3.3%), a Jack Russell terrier (*n* = 1/30; 3.3%), an Australian cattle dog (*n* = 1/30; 3.3%), beagles (*n* = 1/30; 3.3%), an American bully (*n* = 1/30; 3.3%), and a border collie (*n* = 1/30; 3.3%), and bulldog (*n* = 1/30; 3.3%). There was a significant difference in age between Groups 2 and 3 (Table 1) (*p* = 0.0207).

### 3.1. Electrocardiogram

**Figure 1:**
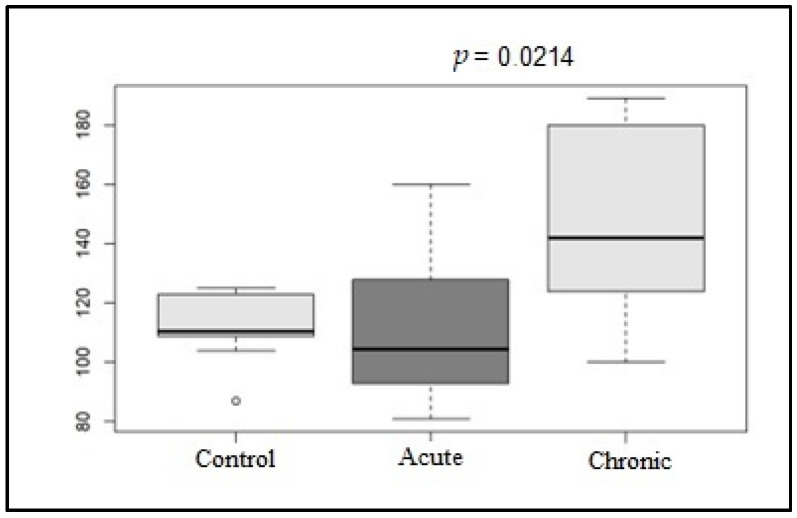
Box plot graphic of the mean HR values (bpm) of the control, acute, and chronic groups on conventional electrocardiogram.

Regarding the predominant rhythm on the ECG, in G1, 50% (5/10) of the dogs had sinus rhythm, 20% (2/10) had sinus arrhythmia, and 30% (3/10) had sinus tachycardia. The mean heart rate for this group was 110.2 beats/min. In G2, 40% (4/10) of the dogs had sinus rhythm, 20% (2/10) had sinus arrhythmia, and 40% (4/10) had sinus tachycardia. The mean heart rate for this group was 145.2 beats/min. In the control group (G3), 90% (9/10) of the dogs had sinus arrhythmia, and 10% (1/10) had sinus rhythm. None had sinus tachycardia. The mean heart rate for G3 was 112.3 beats/min. As shown in Figure 1, a significant difference was observed between the mean heart rates of the G1 and G2 groups (*p* = 0.0214).

In the electrocardiogram, none of the participants in G1 showed arrhythmias during the recording. In contrast, in G2, 20% (2/10) had arrhythmias; one subject had an isolated LV premature beat of origin, while another subject had 49 recorded premature beats during the examination. In G3, one subject had an isolated LV premature beat of origin and first-degree atrioventricular block during the electrocardiogram.

#### 3.1.1. P Wave Analysis

The mean value of the minimum P wave duration (Pmin) was higher in G1, but there was no significant difference between the groups (*p* = 0.82). The mean value of the maximum P wave duration (Pmax) was slightly higher in the acute group, but there was no significant difference (*p* = 0.58).

Regarding P wave dispersion (Pd), the acute group (G1) had higher values compared with the other groups, and a significant difference was observed (*p* = 0.05), as shown in Table 2.

#### 3.1.2. QT Interval Analysis

The mean value of the minimum QT interval duration (QTmin) was higher in the G3 group, and there were significant differences between the G1 and G2 (*p* = 0.0239) groups and between the G2 and G3 (*p* = 0.0019) groups. The mean value of the peak QT interval duration was higher in the G1 group, and there was a significant difference between G1 and G2 (*p* = 0.0262). As for the dispersion of the QT interval, the mean value was higher in the G2 group, and there was a significant difference between G2 and G3 (*p* = 0.0066) (Table 3).

**Figure 2:**
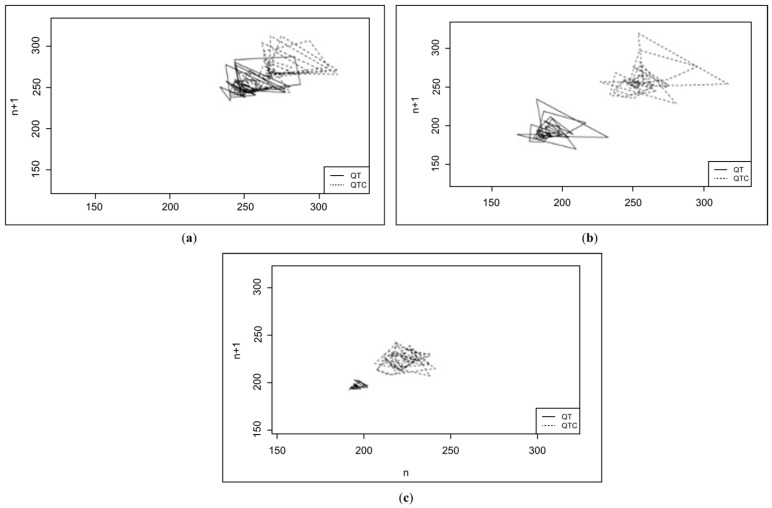
Poincare plot of the dogs in this study: (**a**) the acute phase of the disease group (G1), with TI = 14.36; (**b**) the chronic phase of the disease group (G2), with TI = 11.69; (**c**) the control group, with TI = 5.84.

The mean and median QTc parameters were higher in the G1 and G2 groups in the chronic phase disease group. However, no significant differences were found between the groups in terms of QTc instability parameters. Scatter plots (Poincare plots) were created for each animal in both groups. Each line on the graph represents 49 of the 50 consecutive QT interval values and the corresponding corrected QT, as shown in Figure 2 below.

For the QT and QTc instability parameters, including TI (total instability), STI (short-term instability), and LTI (long-term instability), the mean values of each group were calculated. The mean QT value of G1 was higher than that of G2 and G3. In contrast, the mean QTc value of G2 was higher than that of G1 and G3. However, none of these parameters showed significant differences between the groups.

The QT instability parameters of the control group (G3) were lower than those of the disease groups (G1 and G2), and the mean and median values of TI (*p* = 0.00003), LTI (*p* = 0.0037), and STI (*p* = 0.0003; *p* = 0.0001) in the disease group were significantly higher in the acute phase. The instability parameters showed significant differences between G1 and G3 and between G2 and G3, as shown in Table 4.

#### 3.1.3. Correlation of Age and Heart Rate with Pd, QTd, and QT Instability Parameters

In the present study, the P wave dispersion, QT interval dispersion, and QT instability parameters were also evaluated in relation to age and heart rate since they were the parameters that showed significant differences between the groups (*p* < 0.05). As already mentioned, the animals in the control group were older on average than those in the diseased groups, with a significant difference between G2 and G3 (*p* = 0.0207). At the same time, the mean HR values were higher in the chronic disease group, significantly different from those in the acute disease group (*p* = 0.0214).

Age was weakly negatively correlated with all parameters (Pd, QTd, QT interval, IT, LTI, STI) (r < 0.7). Heart rate was weakly negatively correlated with Pd and QT interval (r < 0.7), while other parameters were weakly positively correlated (r < 0.7).

### 3.2. Holter Analysis

**Figure 3:**
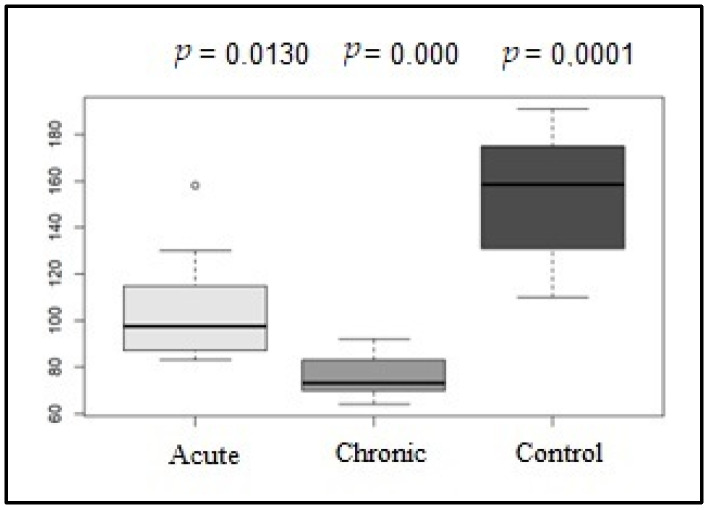
Box plot graphic of the mean heart rate (bpm) values of the acute, control, and chronic groups in the Holter analysis.

After the 24 h Holter analysis, the mean HR was determined in addition to HRV parameters. Regarding the mean HR, the G1 and G2 groups (*p* = 0.013), the G1 and G3 groups (*p* = 0.000), and the G2 and G3 groups (0.0001) showed significant differences, as shown in Figure 3.

Arrhythmias were quantified during the 24 h Holter recordings. Within G3, two animals had premature ventricular beats (2 in one animal and 15 in the second, detached from the left ventricle). In addition, all animals in this group experienced pauses of no more than 1.5 s, mainly during the night.

In the diseased groups (G1 and G2), seven animals had premature ventricular beats, one of which showed 7578 premature ventricular beats during the recording (both isolated and bigeminy, originating from the left ventricle); nine animals had paired or isolated supraventricular arrhythmias; two animals had atrial tachycardia; one patient had second-degree Mobitz type II atrioventricular block; three animals had ventricular escape beats; and only one patient had pauses lasting approximately 2 s. All diseased animals had sinus tachycardia. These observations are consistent with the arrhythmias found in studies of dogs with CME [14].

In the time domain HRV analysis, the SDNN and rMSSD parameters showed significant differences among the groups, while SDANN and pNN50 showed significant differences between G1 and G2 and between G2 and G3, as shown in Table 5. In the frequency domain, high frequency (HF) showed significant differences between G2 and G3; low frequency (LF) showed significant differences between G1 and G2; the LF/HF ratio showed significant differences between G1 and G3 and between G2 and G3; and HRV showed significant differences between G1 and G2 and between G2 and G3, as shown in Table 6.

Correlations were established between the arrhythmia prediction parameters (Pd, QTd, TI, LTI, and STI) and Holter-determined parameters (SDNN, SDANN, rMSSD, pNN50, HF, LF, LF/HF, and VLF) in G1, G2, and G3, but there was no significance between the parameters (*p* > 0.05).

## 4. Discussion

In this study, three heterogeneous groups of dogs were examined, mainly in terms of age, as the mean age of the group of dogs with chronic phase disease was lower than that of the healthy dogs and the group of dogs with acute phase disease. The mean age observed in the G2 group was 2.6 years (range of 4 months to 5.6 years), which is consistent with the results of a retrospective study that examined 19 dogs naturally infected with chronic monocytic ehrlichiosis [15].

According to the literature [16], this situation is not due to a more pronounced immunosuppression in puppies and young dogs but rather to environmental factors, such as a high prevalence of the vector and contact with ticks, which increase the likelihood of early infection. In the cited study, the Botucatu region was identified as an endemic area for the disease, with a prevalence of 40% (28/70) in symptomatic dogs, which explains the higher incidence of primary infection in dogs under one year old [17].

After completing doxycycline treatment, the frequency of arrhythmias on the dog’s electrocardiogram decreased, confirming the effects of acute CME on the cardiovascular system [18].

Furthermore, anemia is one of the most common laboratory findings in CME. Human studies have shown that low hemoglobin concentrations increase sympathetic nerve activity, leading to disruptions in both the temporal and frequency domains of HRV [19,20]. However, there are no studies on livestock, and further investigation of the effects of anemia on ECG parameters and predictors of arrhythmias in dogs is needed.

When evaluating the conventional electrocardiogram, the mean heart rate was significantly higher during the G2 phase, although sinus tachycardia was predominantly observed during the examination. These results are consistent with literature reports [21]. The animals in the control group showed predominantly sinus arrhythmia. As shown in previous studies, this suggests a trend toward increased sympathetic nervous system (SNS) activity in diseased animals [6,7]. In addition, the highest heart rate was observed during G3, indicating that sympathetic nerve activation increases with disease progression.

In severe cases of canine Ehrlichia infection, an inflammatory response syndrome has been documented, which may explain myocardial damage in affected dogs, leading to arrhythmias and elevated troponin levels [7]. Increased inflammation is associated with poor cardiac outcomes and, if persistent, can lead to atrial tissue damage, myocyte necrosis, and fibrosis, ultimately resulting in increased P wave duration and dP [22]. This is promising for evaluating the reduction in the incidence of arrhythmias after CME treatment, or even its prevention, since dogs do not show acute-phase arrhythmias on routine electrocardiograms.

On the electrocardiogram, the P wave represents the electrical activity of the atrial muscle, and an increase in its duration indicates left atrial overload [11]. Although no significant differences were found, the absolute values of Pmin and Pmax were lower in the control group, despite the older age of its members. In addition, as shown in previous studies [22,23], the P wave dispersion values in the control group were lower than those in the diseased groups (G1 and G2).

Although the first group did not show atrial arrhythmias on the electrocardiogram, the acute onset group (G1) had higher Pd values than the chronic onset group (G2). According to the literature [24], Pd was strongly positively correlated with Pmax values (r = 0.702, *p* < 0.001) and weakly positively correlated with age (r = 0.270, *p* < 0.001), and G1 showed higher values for both parameters, which may explain the higher values in the acute phase of the disease rather than in the chronic phase, as expected.

For QTd, the absolute values increased in the chronic group, whereas they decreased in the control group. The control group showed statistically significant differences compared with both the acute and chronic groups. The acute group had higher QT instability parameters, and although these values were very similar to those in the chronic group, there were significant differences between G1 and G3. These results suggest that animals naturally infected with *E. canis* have increased parameters, indicating arrhythmogenic potential. However, although the G2 members had more ventricular arrhythmias, they did not show increased values in the chronic stage of the disease.

The difference in dispersion between the healthy and diseased groups was evident, as shown in the Poincare plots comparing the groups. This supports the hypothesis of heterogeneity in ventricular electrical activity in animals affected by *E. canis*, as all QT instability parameters were higher in these animals than in the healthy group. No significant differences were observed between the acute and chronic animals, despite higher absolute values in the G1 animals.

Recent studies have shown that QT instability parameters are more sensitive and specific in predicting ventricular arrhythmias, whereas QTc parameters failed, as the absolute values in the control group were higher than those in the diseased group [25,26].

According to the literature [27], myocarditis is divided into three stages: the first stage is characterized by initial myocardial damage, the second stage is characterized by autoimmune myocardial damage, and the third stage is characterized by cardiac remodeling processes, with eccentric hypertrophy as the main feature. It is possible that the dogs experienced the initial stage of myocarditis in the acute phase, which could explain the absence of supraventricular and ventricular arrhythmias on conventional electrocardiograms. However, this cannot be confirmed with certainty, as troponin I levels were not measured in this group, and histopathological examination, which is considered the gold standard for diagnosing myocarditis, was not performed in either group. There is a lack of studies on myocarditis in dogs, especially those associated with electrocardiographic parameters and arrhythmogenic studies; however, arrhythmic sudden cardiac death in dogs and has been mainly related to arrhythmia secondary to myocardial diseases, such as dilated cardiomyopathy (DCM), arrhythmogenic cardiomyopathy hypertrophy, and myocarditis, which act as anatomical arrhythmic substrates [28]. Currently, in the literature, echocardiographic examination for the diagnosis of endocarditis and myocarditis in dogs (Szalus-Jordanow et al., 2021) has been explored [29].

The parameters evaluated by Holter monitoring over the entire time frame were superior in the control group than in the diseased groups (G1 and G2) and showed significant differences between them, demonstrating a balance between sympathetic and parasympathetic activity throughout the day.

As highlighted in a recent study [6], the parameters related to the parasympathetic system (rMSSD and pNN50) were lower in the diseased group. Moreover, both parameters showed significant differences between healthy animals and animals in the chronic phase of the disease, as well as between dogs in the acute and chronic phases, highlighting the dominance of sympathetic activity in dogs with CME.

The LF/HF ratio reflects the sympathetic–vagal balance, so an increased ratio indicates less vagal activation [30]. The results of this parameter in the control group were similar to those of studies evaluating HRV parameters in healthy dogs [31,32].

According to the literature [31], puppies naturally show a dominance of sympathetic activity and, therefore, have lower values in the time domain. In addition, dogs older than 8 years tend to show a natural decrease in parameters related to parasympathetic regulation.

Within the groups, the dogs in the chronic phase of the disease had a lower mean age (2.6 years), which may further support the dominance of sympathetic activity in this group. In contrast, the control group had a higher mean age (5.6 years) and parameters, suggesting a greater predominance of parasympathetic regulation compared to the other groups.

When all groups were analyzed for HRV parameters and predictors of arrhythmias, no strong evidence was found for a correlation between these parameters.

## 5. Conclusions

In this study, mean values of Pd and QTd were observed, indicating that the animals with CME were prone to supraventricular and ventricular arrhythmias. Despite the absence of supraventricular and ventricular arrhythmias on the ECG in this group, the instability parameters showed worsened values during the acute phase of the disease. This suggests the presence of arrhythmogenic activity in dogs naturally infected with *E. canis*, even if the parameters do not worsen with the progression of the disease.

In addition, after analyzing HRV parameters, it was confirmed that the sympathetic activity in the diseased animals was dominant, resulting in a decrease in parasympathetic activity in the G1 and G2 groups compared to the healthy animals.

This study highlights the importance of evaluating the ECG and arrhythmia markers in the diagnosis and treatment of CME.

## Figures and Tables

**Table 1:** Group characterization in the present study.

Variables/Groups	G1 (Acute)	G2 (Chronic)	G3 (Control)
Age (years)	4.71 ^BC^	2.66 ^AC^	5.67 ^Ab^
Weight (kg)	18.00 ^DF^	15.03 ^DE^	23.01 ^EF^
Male	4/30 (13.3%)	7/30 (23.3%)	7/30 (23.3%)
Female	6/30 (20%)	3/30 (10%)	3/30 (10%)

Different superscript letters (uppercase and lowercase) indicate significant differences (*p* ≤ 0.05), while identical letters signify no significant difference in the parameter among the groups.

**Table 2:** Indices of the P wave: Pmax (maximum duration of P wave), Pmin (minimum duration of P wave), and Pd (P wave dispersion).

	Pmax			Pmin			Pd	
G1 (Acute)	G2 (Chronic)	G3 (Control)	G1 (Acute)	G2 (Chronic)	G3 (Control)	G1 (Acute)	G2 (Chronic)	G3 (Control)
52.67 ± 4.76 ^A^	48.83 ± 5.33 ^A^	46.53 ± 4.75 ^A^	42.03 ± 3.921 ^B^	40.80 ± 3.69 ^B^	39.67 ± 5.17 ^B^	10.56 ± 3.16 ^DE^	7.80 ± 2.55 ^cd^	6.90 ± 2.87 ^Cc^

Different superscript letters (uppercase and lowercase) indicate significant differences (*p* ≤ 0.05), while identical letters signify no significant difference in the parameter among the groups.

**Table 3:** Indices of the QT interval: QTmax (maximum duration of QT interval), QTmin (minimum duration of QT interval), and QTd (QT interval dispersion).

	QTmax			QTmin			QTd	
G1 (Acute)	G2 (Chronic)	G1 (Control)	G1 (Acute)	G2 (Chronic)	G3 (Control)	G1 (Acute)	G2 (Chronic)	G3 (Control)
211.73 ± 15.12 ^aB^	193.70 ± 16.58 ^AC^	208.16 ± 11.46 ^BC^	193.13 ± 13.92 ^EF^	175.06 ± 16.91 ^de^	199.67 ± 11.80 ^DF^	18.13 ± 10.36 ^HI^	18.80 ± 4.64 ^gI^	11.46 ± 10.13 ^GH^

Different superscript letters (uppercase and lowercase) indicate significant differences (*p* ≤ 0.05), while identical letters signify no significant difference in the parameter among the groups.

**Table 4:** Indices of the QT interval instability: TI (total instability), LTI (long-term instability), and STI (short-term instability).

	TI			LTI			STI	
G1 (Acute)	G2 (Chronic)	G3 (Control)	G1 (Acute)	G2 (Chronic)	G3 (Control)	G1 (Acute)	G2 (Chronic)	G3 (Control)
10.548 ± 2.525 ^BC^	9.900 ± 1.886 ^AC^	6.966 ± 0.972 ^ab^	7.658 ± 1.622 ^DF^	7.232 ± 0.823 ^EF^	6.125 ± 0.609 ^dc^	5.754 ± 1.730 ^GI^	5.415 ± 1.853 ^HI^	2.329 ± 0.698 ^GI^

Different superscript letters (uppercase and lowercase) indicate significant differences (*p* ≤ 0.05), while identical letters signify no significant difference in the parameter among the groups.

**Table 5:** Heart rate variability parameters (time domain) in the dogs with chronic and acute Ehrlichiosis and the control dogs.

SDNN			rMSSD		
G1 (Acute)	G2 (Chronic)	G3 (Control)	G1 (Acute)	G2 (Chronic)	G3 (Control)
191.90 ± 78.069 ^Ac^	70.5 ± 46.869 ^ab^	286.86 ± 59.87 ^BC^	157.900 ± 82.447 ^Df^	45.600 ± 37.757 ^de^	243.533 ± 74.607 ^EF^
**SDANN**				**pNN50**	
**G1** **(Acute)**	**G2** **(Chronic)**	**G3** **(Control)**	**G1** **(Acute)**	**G2** **(Chronic)**	**G3** **(Control)**
129.8 ± 61.984 ^GI^	49.4 ± 29.75 ^gH^	166.200 ± 25.25 ^hI^	45.021 ± 21.512 ^JL^	10.425 ± 12.673 ^jk^	61.972 ± 12.065 ^KL^

Different superscript letters (uppercase and lowercase) indicate significant differences (*p* ≤ 0.05), while identical letters signify no significant difference in the parameter among the groups.

**Table 6:** Heart rate variability parameters (frequency domain) in the dogs with chronic and acute Ehrlichiosis and the control dogs.

	HF			LF			LF/HF			VLF	
G1 (Acute)	G2 (Chronic)	G3 (Control)	G1 (Acute)	G2 (Chronic)	G3 (Control)	G1 (Acute)	G3 (Control)	G2 (Chronic)	G1 (Acute)	G2 (Chronic)	G3 (Control)
3112.60 ± 2151.86 ^BC^	176.65 ± 211.747 ^AB^	12,648.51 ± 9293.92 ^aC^	1624.85± 961.72 ^DE^	181.90 ± 144.98 ^dF^	3228.90 ± 4395.43 ^EF^	1.10 ± 0.86 ^GI^	0.21 ± 0.20 ^gh^	2.82 ± 4.45 ^HI^	1910.85 ± 1720.41 ^JL^	261 ± 259.11 ^jk^	7467.59 ± 10,495.73 ^KL^

Different superscript letters (uppercase and lowercase) indicate significant differences (*p* ≤ 0.05), while identical letters signify no significant difference in the parameter among the groups.

## Data Availability

The data supporting the reported results can be found at Repositório Institucional Unesp: https://repositorio.unesp.br/entities/publication/99c66ce3-0fb5-44fd-8a38-f469e4d9bd36 (accessed on 27 April 2023).

## Abbreviations

The following abbreviations are used in this manuscript:

CEUA	Ethics Committee on the Use of Animals
CME	canine monocytic ehrlichiosis
HF	high-frequency component of HRV
HRV	heart rate variability
LF	low-frequency component of HRV
LF/HF	low-frequency/High-frequency ratio
LTI	long-term instability
PCR	polymerase chain reaction
Pd	P wave dispersion
Pd	P wave dispersion
Pmax	largest P wave value
Pmin	smallest P wave value
pNN50	percentage of adjacent R-R intervals with duration differences greater than 50 ms
QTc	QT corrected
QTd	QT interval dispersion
QTd	largest QT interval value
QTm	QT mean
QTmax	largest QT interval value
QTmin	smallest QT interval value
QTv	QT variance
rMSSD	root mean square of successive differences in consecutive R–R intervals
rMSSD	root mean square of successive differences in consecutive R–R intervals
RR or NN	interval between two R waves of the electrocardiogram
SDANN	standard deviation of the averages of normal R-R intervals every 5 min
SDNN	standard deviation of normal RR or NN intervals over a specified period
STI	short-term instability
TI	total instability
